# CT and MRI radiomics of bone and soft-tissue sarcomas: a systematic review of reproducibility and validation strategies

**DOI:** 10.1186/s13244-021-01008-3

**Published:** 2021-06-02

**Authors:** Salvatore Gitto, Renato Cuocolo, Domenico Albano, Francesco Morelli, Lorenzo Carlo Pescatori, Carmelo Messina, Massimo Imbriaco, Luca Maria Sconfienza

**Affiliations:** 1 Dipartimento di Scienze Biomediche per la Salute, Università degli Studi di Milano, Via Riccardo Galeazzi 4, 20161 Milan, Italy; 2 Dipartimento di Medicina Clinica e Chirurgia, Università degli Studi di Napoli “Federico II”, Naples, Italy; 3 Laboratory of Augmented Reality for Health Monitoring (ARHeMLab), Dipartimento di Ingegneria Elettrica e delle Tecnologie dell’Informazione, Università degli Studi di Napoli “Federico II”, Naples, Italy; 4 IRCCS Istituto Ortopedico Galeazzi, Milan, Italy; 5 Sezione di Scienze Radiologiche, Dipartimento di Biomedicina, Neuroscienze e Diagnostica Avanzata, Università degli Studi di Palermo, Palermo, Italy; 6 ASST Grande Ospedale Metropolitano Niguarda, Milan, Italy; 7 Assistance Publique - Hôpitaux de Paris (AP-HP), Service d’Imagerie Médicale, CHU Henri Mondor, Créteil, France; 8 Dipartimento di Scienze Biomediche Avanzate, Università degli Studi di Napoli “Federico II”, Naples, Italy

**Keywords:** Artificial intelligence, Radiomics, Sarcoma, Texture analysis

## Abstract

**Background:**

Feature reproducibility and model validation are two main challenges of radiomics. This study aims to systematically review radiomic feature reproducibility and predictive model validation strategies in studies dealing with CT and MRI radiomics of bone and soft-tissue sarcomas. The ultimate goal is to promote achieving a consensus on these aspects in radiomic workflows and facilitate clinical transferability.

**Results:**

Out of 278 identified papers, forty-nine papers published between 2008 and 2020 were included. They dealt with radiomics of bone (*n* = 12) or soft-tissue (*n* = 37) tumors. Eighteen (37%) studies included a feature reproducibility analysis. Inter-/intra-reader segmentation variability was the theme of reproducibility analysis in 16 (33%) investigations, outnumbering the analyses focused on image acquisition or post-processing (*n* = 2, 4%). The intraclass correlation coefficient was the most commonly used statistical method to assess reproducibility, which ranged from 0.6 and 0.9. At least one machine learning validation technique was used for model development in 25 (51%) papers, and K-fold cross-validation was the most commonly employed. A clinical validation of the model was reported in 19 (39%) papers. It was performed using a separate dataset from the primary institution (i.e., internal validation) in 14 (29%) studies and an independent dataset related to different scanners or from another institution (i.e., independent validation) in 5 (10%) studies.

**Conclusions:**

The issues of radiomic feature reproducibility and model validation varied largely among the studies dealing with musculoskeletal sarcomas and should be addressed in future investigations to bring the field of radiomics from a preclinical research area to the clinical stage.

## Key points

- Radiomic studies focused on CT and MRI of musculoskeletal sarcomas were reviewed.
- Feature reproducibility analysis and model validation strategies varied largely among these studies.
- Radiomic feature reproducibility was assessed in less than half of the studies.
- Only 10% of the studies included an independent clinical validation of the model.

## Background

Bone and soft-tissue primary malignant tumors or sarcomas are rare entities with several histological subtypes, and each has an incidence < 1/100,000/year [1, 2]. Among them, osteosarcoma is the most common sarcoma of the bone. Along with Ewing sarcoma, it has a higher incidence in the second decade of life, while chondrosarcoma is the most prevalent bone sarcoma in adulthood [1]. The most frequent soft-tissue sarcomas are liposarcoma and leiomyosarcoma [2]. Due to the rarity of these diseases, bone and soft-tissue sarcomas are managed in tertiary sarcoma centers according to current guidelines [1, 2]. Both biopsy and imaging integrate clinical data prior to the beginning of any treatment, with the former representing the reference standard for preoperative diagnosis [1, 2]. However, biopsy may be inaccurate in large, heterogeneous tumors due to sampling errors, and, in turn, inaccurate diagnosis may lead to inadequate treatment and subsequent need for further interventions, with increased morbidity. Additionally, the risk of biopsy tract contamination remains a concern. Imaging already plays a pivotal role in the assessment of bone and soft-tissue sarcomas. Magnetic resonance imaging (MRI) and computed tomography (CT) are employed for local and general staging, respectively [1, 2]. These modalities may certainly benefit from new imaging-based tools such as those based on radiomics, which may potentially provide additional information regarding both diagnosis and prognosis noninvasively [3].

The term “radiomics” derives from a combination of “radio,” referring to medical images and “omics,” which indicates the analysis of high amounts of data representing an entire set of some kind, like genome (genomics) and proteome (proteomics) [3]. Therefore, “radiomics” includes extraction and analysis of large numbers of quantitative parameters, known as radiomic features, from medical images [4]. This technique has recently gained much attention in oncologic imaging as it can potentially quantify tumor heterogeneity, which can be challenging to capture by means of qualitative imaging assessment or sampling biopsies. Particularly, radiomic studies to date have focused on discriminating tumor grades and types before treatment, monitoring response to therapy and predicting outcome [5].

Despite its great potential as a noninvasive tumor biomarker, radiomics still faces challenges preventing its clinical implementation. Two main initiatives have addressed methodological issues of radiomic studies to bridge the gap between academic endeavors and real-life application. In 2017, Lambin et al. proposed the Radiomics Quality Score that details the sequential steps to follow in radiomic pipelines and offers a tool to assess methodological rigor in their implementation [6]. In 2020, the Image Biomarkers Standardization Initiative produced and validated reference values for radiomic features, which enable verification and calibration of different software for radiomic feature extraction [7]. However, numerous challenges still remain to ensure clinical transferability of radiomics. As radiomics is essentially a two-step approach consisting of data extraction and analysis, in the first step (i.e., data extraction), the main challenge is reproducibility of radiomic features, which can be influenced by image acquisition parameters, region of interest segmentation technique and image post-processing technique [8, 9]. In the second step (i.e., data analysis), models can be built upon either conventional statistical methods or machine learning algorithms with the aim of predicting the diagnosis or outcome of interest. In either case, the main challenge is model validation [9].

The challenges of reproducibility and validation strategies in radiomics have been recently addressed in a review focusing on renal masses [10]. The aim of our study is to systematically review radiomic feature reproducibility and predictive model validation strategies in studies dealing with CT and MRI radiomics of bone and soft-tissue sarcomas. The ultimate goal is to promote and facilitate achieving a consensus on these aspects in radiomic workflows.

## Methods

### Reviewers

No Local Ethics Committee approval was needed for this systematic review. Literature search, study selection, and data extraction were performed independently by two recently boarded radiologists with experience in musculoskeletal tumors and radiomics (S.G. and F.M.). In case of disagreement, agreement was achieved by consensus of these two readers and a third reviewer with radiology specialty and doctorate in artificial intelligence and radiomics (R.C.). The Preferred Reporting Items for Systematic reviews and Meta-Analyses (PRISMA) guidelines [11] were followed.

### Literature search

An electronic literature search was conducted on EMBASE (Elsevier) and PubMed (MEDLINE, U.S. National Library of Medicine and National Institutes of Health) databases for articles published up to December 31, 2020, and dealing with CT and MRI radiomics of bone and soft-tissue sarcomas. A controlled vocabulary was adopted using medical subject headings in PubMed and the thesaurus in EMBASE. Search syntax was built by combining search terms related to two main domains, namely “musculoskeletal sarcomas” and “radiomics.” The exact search query was: (“sarcoma”/exp OR “sarcoma”) AND (“radiomics”/exp OR “radiomics” OR “texture”/exp OR “texture”). Studies were first screened by title and abstract, and then, the full text of eligible studies was retrieved for further review. The references of selected publications were checked for additional publications to include.

### Inclusion and exclusion criteria

Inclusion criteria were: (1) original research papers published in peer-reviewed journals; (2) focus on CT or MRI radiomics-based characterization of sarcomas located in bone and soft tissues for either diagnosis- or prognosis-related tasks; (3) statement that local ethics committee approval was obtained, or ethical standards of the institutional or national research committee were followed.

Exclusion criteria were: (1) papers not dealing with mass characterization, such as those focused on computer-assisted diagnosis and detection systems; (2) papers dealing with head and neck, retroperitoneal or visceral sarcomas; (3) animal, cadaveric or laboratory studies; (4) papers not written in English language.

### Data extraction

Data were extracted to a spreadsheet with a drop-down list for each item, as defined by the first author, grouped into three main categories, namely baseline study characteristics, radiomic feature reproducibility strategies, and predictive model validation strategies. Items regarding baseline study characteristics included first author’s last name, year of publication, study aim, tumor type, study design, reference standard, imaging modality, database size, use of public data, segmentation process, and segmentation style. Those concerning radiomic feature reproducibility strategies included reproducibility assessment based on repeated segmentations, reproducibility assessment related to acquisition or post-processing techniques, statistical method used for reproducibility analysis, and cut-off or threshold used for reproducibility analysis. Finally, data regarding predictive model validation strategies included the use of machine learning validation techniques, clinical validation performed on a separate internal dataset, and clinical validation performed on an external or independent dataset.

## Results

### Baseline study characteristics

**Fig. 1:**
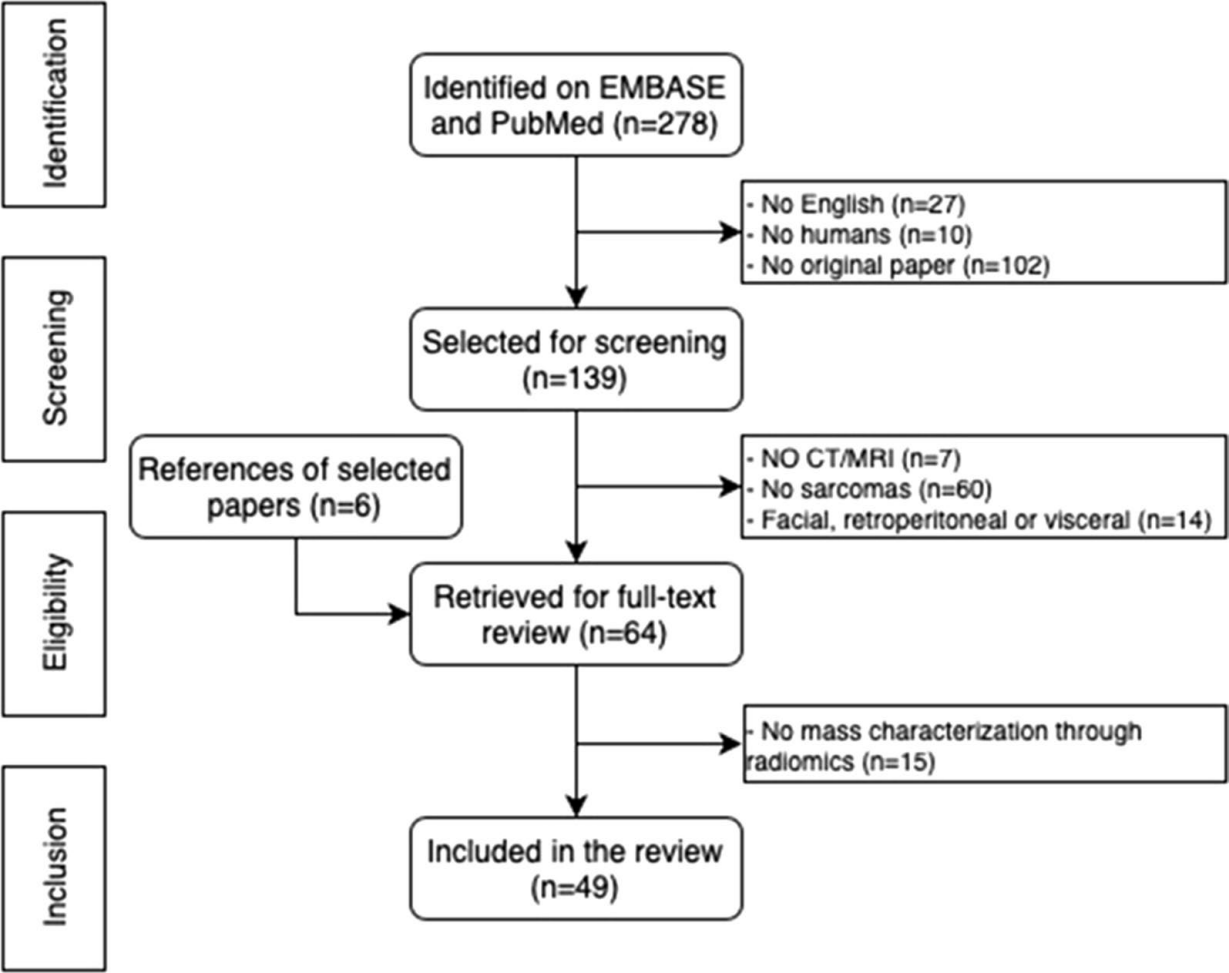
PRISMA (Preferred Reporting Items for Systematic reviews and Meta-Analyses) flowchart of systematic identification, screening, eligibility and inclusion information from retrieved studies

A flowchart illustrating the literature search process is presented in Fig. 1. After screening 278 papers and applying our eligibility criteria, 49 papers were included in this systematic review. Tables 1 and 2 detail the characteristics of papers dealing with radiomics of bone (*n* = 12) and soft-tissue (*n* = 37) tumors, respectively.

**Table 1:** Characteristics of the papers dealing with bone sarcomas included in the systematic review

First author	Year	Aim	Tumor	Design	Reference standard	Modality	Database size (*n*)	Public data	Segmentation	
									Process	Style
Baidya Kayal [50]	2020	Therapy response	Osteosarcoma	Prospective	Histology	MRI	32	No	Manual	3D
Chen [29]	2020	Local relapse Metastatic relapse	Osteosarcoma	Retrospective	Histology Imaging	MRI	93	No	Manual	2D without MS
Dai [45]	2020	Histotype	Ewing sarcoma Osteosarcoma	Retrospective	Histology	MRI	66	No	Manual	2D without MS
Fritz [13]	2018	Benign versus malignant Grading	Chondroma Chondrosarcoma	Retrospective	Histology Imaging	MRI	116	No	Manual	2D without MS
Gitto [46]	2020	Grading	Chondrosarcoma	Retrospective	Histology	MRI	58	No	Manual	2D without MS
Li [32]	2019	Histotype	Chondrosarcoma Chordoma	Retrospective	Histology	MRI	210	No	Manual	3D
Lin [15]	2020	Therapy response	Osteosarcoma	Retrospective	Histology	CT	191	No	Manual	3D
Lisson [12]	2018	Benign versus malignant	Chondroma Chondrosarcoma	Retrospective	Histology Imaging	MRI	22	No	Semiautomatic	3D
Wu [16]	2018	Survival	Osteosarcoma	Retrospective	Follow-up	CT	150	No	Manual	3D
Yin [38]	2020	Local relapse	Chondrosarcoma	Retrospective	Histology Imaging	MRI	103	No	Manual	3D
Yin [28]	2019	Histotype	Chordoma Giant cell tumor	Retrospective	Histology	CT	95	No	Manual	3D
Zhao [22]	2019	Survival	Osteosarcoma	Retrospective	Follow-up	MRI	112	No	Manual	3D

*MS* multiple sampling

**Table 2:** Characteristics of the papers dealing with soft-tissue sarcomas included in the systematic review

First author	Year	Aim	Tumor	Design	Reference standard	Modality	Database size (*n*)	Public data	Segmentation	
									Process	Style
Corino [52]	2018	Grading	Multiple sarcoma histotypes	Retrospective	Histology	MRI	19	No	Manual	3D
Crombé [39]	2020	Metastatic relapse Survival	Liposarcoma	Retrospective	Histology Follow-up	MRI	35	No	Manual	3D
Crombé [42]	2020	Metastatic relapse Survival	Multiple sarcoma histotypes	Retrospective	Histology Follow-up	MRI	50	No	Manual	3D
Crombé [30]	2019	Therapy response	Multiple sarcoma histotypes	Prospective	Histology Imaging	MRI	25	No	Manual	3D
Crombé [47]	2019	Therapy response	Multiple sarcoma histotypes	Retrospective	Histology	MRI	65	No	Manual	3D
Crombé [63]	2020	Therapy response Survival	Liposarcoma	Retrospective	Histology Follow-up	MRI	21	No	Manual	3D
Crombé [36]	2020	Therapy response Survival	Desmoid tumor	Retrospective	Imaging Follow-up	MRI	42	No	Manual	3D
Crombé [31]	2020	Metastatic relapse Survival	Multiple sarcoma histotypes	Retrospective	Histology Follow-up	MRI	70	No	Manual	3D
Gao [33]	2020	Therapy response	Multiple sarcoma histotypes	Prospective	Histology	MRI	30	No	Manual	3D
Hayano [21]	2015	Survival	Multiple sarcoma histotypes	Prospective	Follow-up	CT	20	No	Manual	2D without MS
Hong [64]	2020	Grading	Multiple sarcoma histotypes	Retrospective	Histology	MRI	42	No	Manual	3D
Juntu [48]	2010	Benign versus malignant	Multiple benign/ malignant histotypes	Retrospective	Histology	MRI	135	No	Manual	2D with MS
Kim [65]	2017	Benign versus malignant	Multiple benign/ malignant histotypes	Retrospective	Histology	MRI	40	No	Manual	3D
Leporq [19]	2020	Benign versus malignant	Lipoma Liposarcoma	Retrospective	Histology	MRI	81	No	Manual	2D without MS
Malinauskaite [23]	2020	Benign versus malignant	Lipoma Liposarcoma	Retrospective	Histology	MRI	38	No	Semiautomatic	3D
Martin-Carreras [34]	2019	Benign versus malignant	Myxoma Myxofibrosarcoma	Retrospective	Histology	MRI	56	No	Manual	3D
Mayerhoefer [57]	2008	Benign versus malignant	Multiple benign/ malignant histotypes Non-tumoral lesions	Retrospective	Histology	MRI	58	No	Manual	2D with MS
Meyer [66]	2019	Proliferation index	Multiple sarcoma histotypes	Retrospective	Histology	MRI	29	No	Manual	2D without MS
Peeken [25]	2019	Grading Survival	Multiple sarcoma histotypes	Retrospective	Histology Follow-up	MRI	225	No	Manual	3D
Peeken [27]	2019	Grading Survival	Multiple sarcoma histotypes	Retrospective	Histology Follow-up	CT	221	Yes	Manual	3D
Pressney [14]	2020	Benign versus malignant	Lipoma Liposarcoma	Retrospective	Histology	MRI	60	No	Manual	2D without MS
Spraker [51]	2019	Survival	Multiple sarcoma histotypes	Retrospective	Follow-up	MRI	226	No	Manual	3D
Tagliafico [26]	2019	Local relapse	Multiple sarcoma histotypes	Prospective	Histology Imaging	MRI	19	No	Manual	2D with MS
Thornhill [49]	2014	Benign versus malignant	Lipoma Liposarcoma	Retrospective	Histology	MRI	44	No	Semiautomatic	3D
Tian [35]	2020	Metastatic relapse	n/a	Retrospective	Histology Imaging	MRI	77	No	Manual	3D
Tian [67]	2015	Therapy response Survival	Multiple sarcoma histotypes	Prospective	Histology Follow-up	CT	20	No	Manual	2D without MS
Timbergen [20]	2020	Histotype	Desmoid tumor Multiple sarcoma histotypes	Retrospective	Histology	MRI	203	No	Manual	3D
Vallières [55]	2015	Metastatic relapse	Multiple sarcoma histotypes	Retrospective	Histology Imaging	MRI	51	Yes	Manual	3D
Vallières [68]	2017	Metastatic relapse	Multiple sarcoma histotypes	Retrospective	Histology Imaging	MRI	30	Yes	Manual	3D
Vos [40]	2019	Benign versus malignant	Lipoma Liposarcoma	Retrospective	Histology	MRI	116	No	Semiautomatic	3D
Wang [41]	2020	Grading	Multiple sarcoma histotypes	Retrospective	Histology	MRI	113	No	Manual	3D
Wang [43]	2020	Benign versus malignant	Multiple benign/ malignant histotypes	Retrospective	Histology	MRI	206	No	Manual	3D
Wang [24]	2020	Benign versus malignant	Multiple benign/ malignant histotypes	Retrospective	Histology	MRI	91	No	Manual	3D
Wu [18]	2020	Benign versus malignant	Multiple benign/ malignant histotypes	Retrospective	Histology	CT	49	No	Manual	2D without MS
Xiang [17]	2019	Grading	Multiple sarcoma histotypes	Retrospective	Histology	MRI	67	No	Manual	2D without MS
Xu [37]	2020	Grading	Multiple sarcoma histotypes	Retrospective	Histology	MRI	105	No	Manual	3D
Zhang [44]	2019	Grading	Multiple sarcoma histotypes	Retrospective	Histology	MRI	37	No	Manual	3D

*MS* multiple sampling

All studies were published between 2008 and 2020. Twenty-three out of 49 investigations (47%) were published in 2020, 14 (29%) in 2019, 4 (8%) in 2018, and 8 (16%) between 2008 and 2017. The design was prospective in 6 studies (12%) and retrospective in the remaining 43 (88%). The imaging modality of choice was MRI in 42 (86%), including one or multiple MRI sequences, and CT in 7 (14%) cases. The median size of the database was 60 patients (range 19–226). Public data were used only in 3 (6%) studies.

The research was aimed at predicting either diagnosis or prognosis, as follows: benign versus malignant tumor discrimination (*n* = 14); grading (*n* = 10); tumor histotype discrimination (*n* = 4); proliferation index Ki67 expression (*n* = 1); survival (*n* = 12); response to therapy, either chemotherapy or radiotherapy (*n* = 8); local and/or metastatic relapse (*n* = 9). It should be noted that the aim was twofold in some studies, as detailed in Tables 1 and 2. In those focused on diagnosis-related tasks, including benign versus malignant discrimination, grading, tumor histotype discrimination, and proliferation index expression, histology was the reference standard in all cases excepting benign lesions diagnosed on the basis of stable imaging findings over time in two papers [12, 13]. In studies focused on prediction of response to chemotherapy or radiotherapy, the reference standard was histology if lesions were surgically treated, based on the percentage of viable tumor and necrosis relative to the surgical tissue specimen, or consistent imaging findings if lesions were not operated. In studies focused on prediction of tumor relapse, the diagnosis was based on histology or consistent imaging findings, as the reference standard. In studies dealing with survival prediction, survival was assessed based on follow-up.

Regarding segmentation, the process was performed manually in 45 (92%) studies and semiautomatically in 4 (8%) studies. In no case, the segmentation process was fully automated. The following segmentation styles were identified: 2D without multiple sampling in 11 (23%) studies; 2D with multiple sampling in 3 (6%); 3D in 35 (71%). Of note, a single slice showing maximum tumor extension was chosen in all studies employing 2D segmentation without multiple sampling, excepting one case [14] where it was chosen based on signal intensity homogeneity.

### Reproducibility strategies

Eighteen (37%) of the 49 studies included a reproducibility analysis of the radiomic features in their workflow. In 16 (33%) investigations [13, 15–29], the reproducibility of radiomic features was assessed on the basis of repeated segmentations performed by different readers and/or the same reader at different time points. Two (4%) studies presented an analysis to assess the reproducibility based on different acquisition [30] or post-processing [31] techniques. Of note, segmentations were validated by a second experienced reader in 15 studies [12, 32–45] without, however, addressing the issue of radiomic feature reproducibility.

The intraclass correlation coefficient (ICC) was the statistical method used in most of the papers reporting a reproducibility analysis [13, 15–18, 20, 22–25, 27–29, 31]. ICC threshold ranged between 0.6 [13] and 0.9 [22] for reproducible features. The following statistical methods were used less commonly: analysis of variance [30, 31]; Cronbach alpha statistic [26]; Pearson correlation coefficient [19], and Spearman correlation coefficient [21].

### Validation strategies

**Fig. 2:**
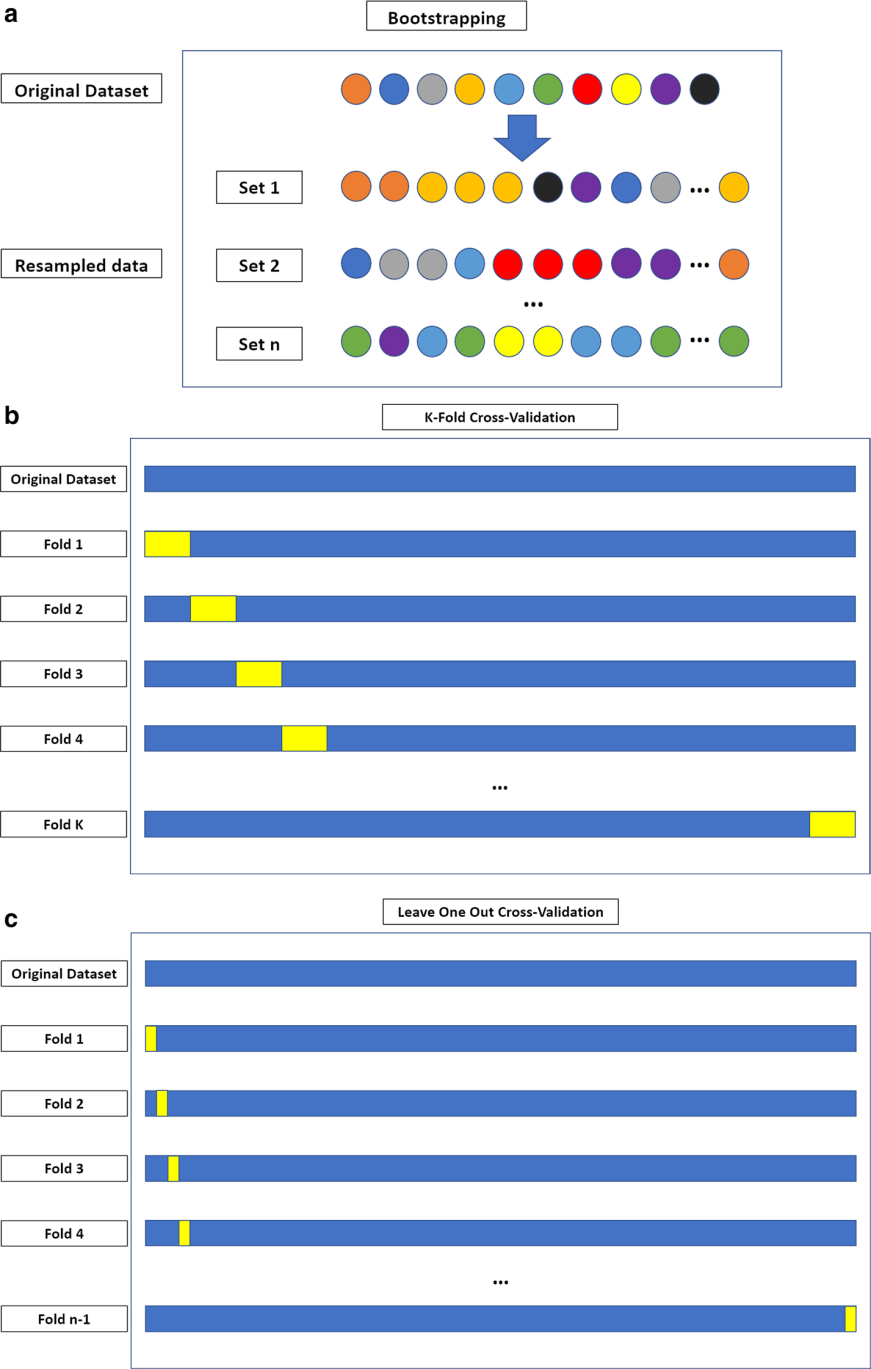

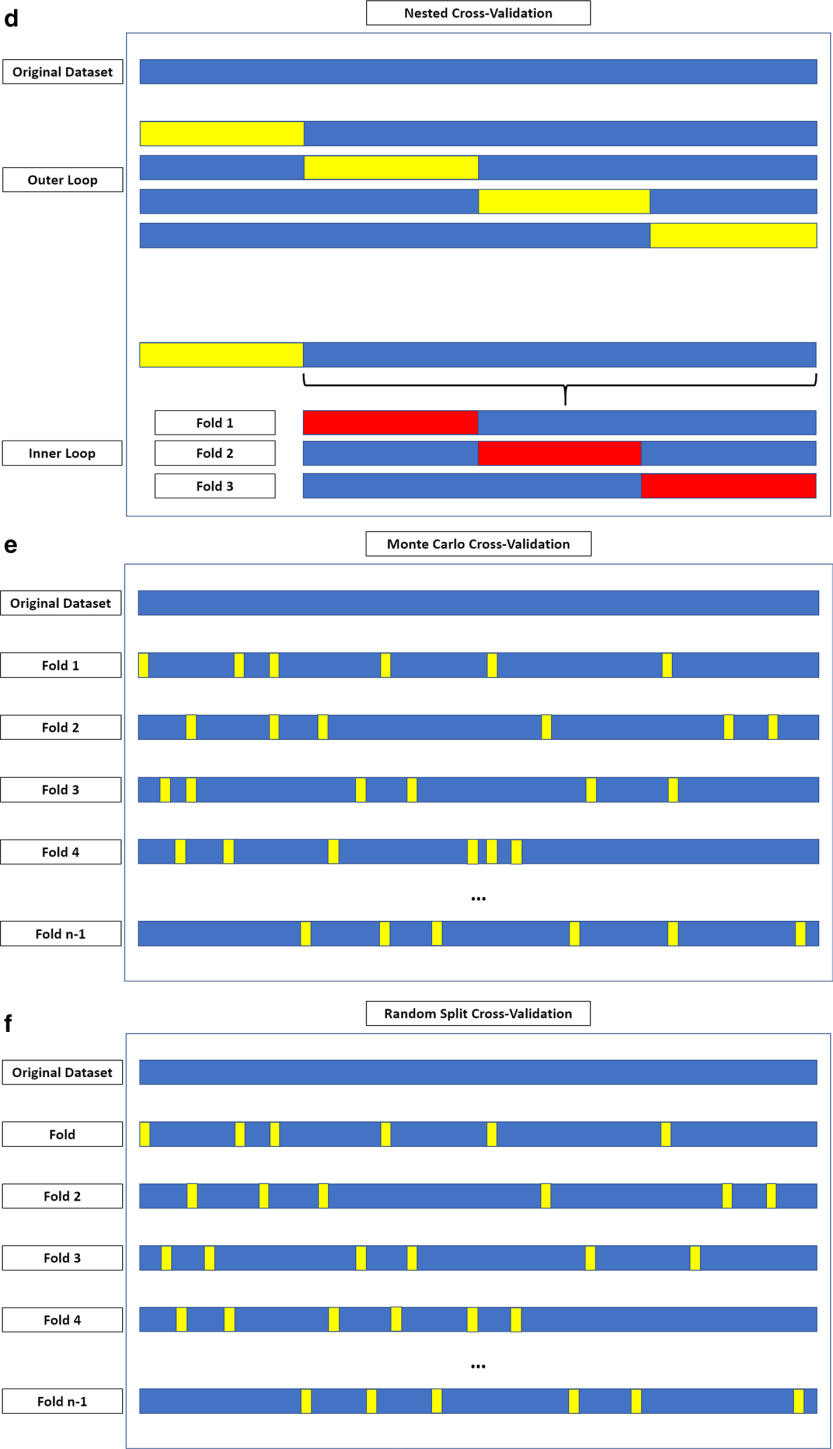

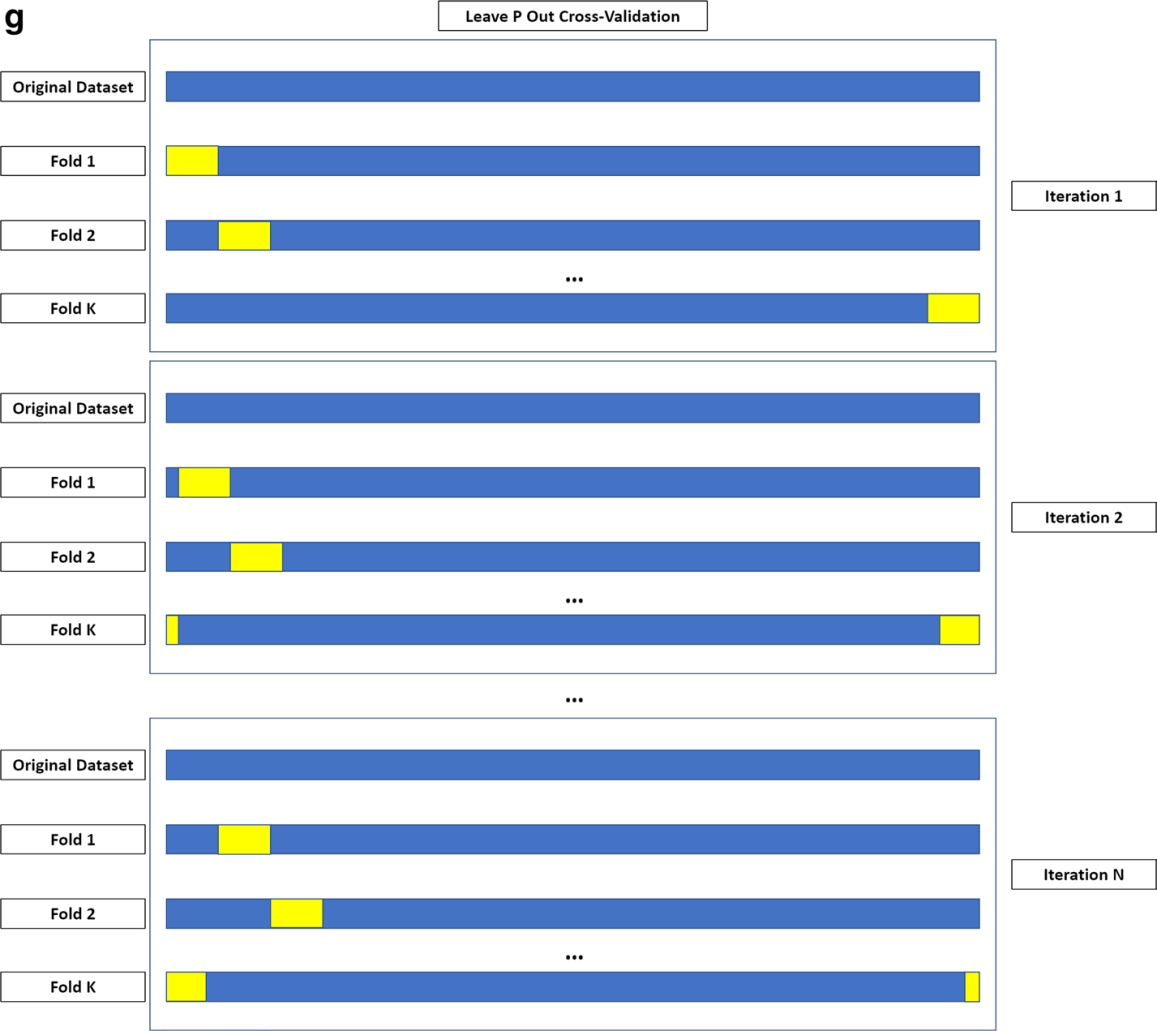
Overview of machine learning validation techniques. **a** Bootstrapping is based on resampling with replacement, allowing to create *n* datasets from an original sample. These may include any number of copies of a specific instance from the original case, even none. **b** K-fold cross-validation is based on dividing the dataset in *k* parts, using each in turn as the validation set and the remaining as the training data. **c** In leave-one-out cross-validation, each instance in the dataset is used for model validation, using the remaining for model training. **d** In nested cross-validation, two loops of validation take place. The training data from each outer loop undergo an additional K-fold cross-validation. The figure depicts a fourfold outer loop paired with a threefold inner loop. In (**e**) Monte Carlo and (**f**) random-split cross-validation, the folds are not made up of contiguous data but from random sampling of the entire dataset. During the first, a sample may appear in multiple folds, which is not possible in random-split cross-validation. **g** In leave-P-out cross-validation, the K-fold cross-validation process is iterated to obtain all possible folding splits for the data

**Fig. 3:**
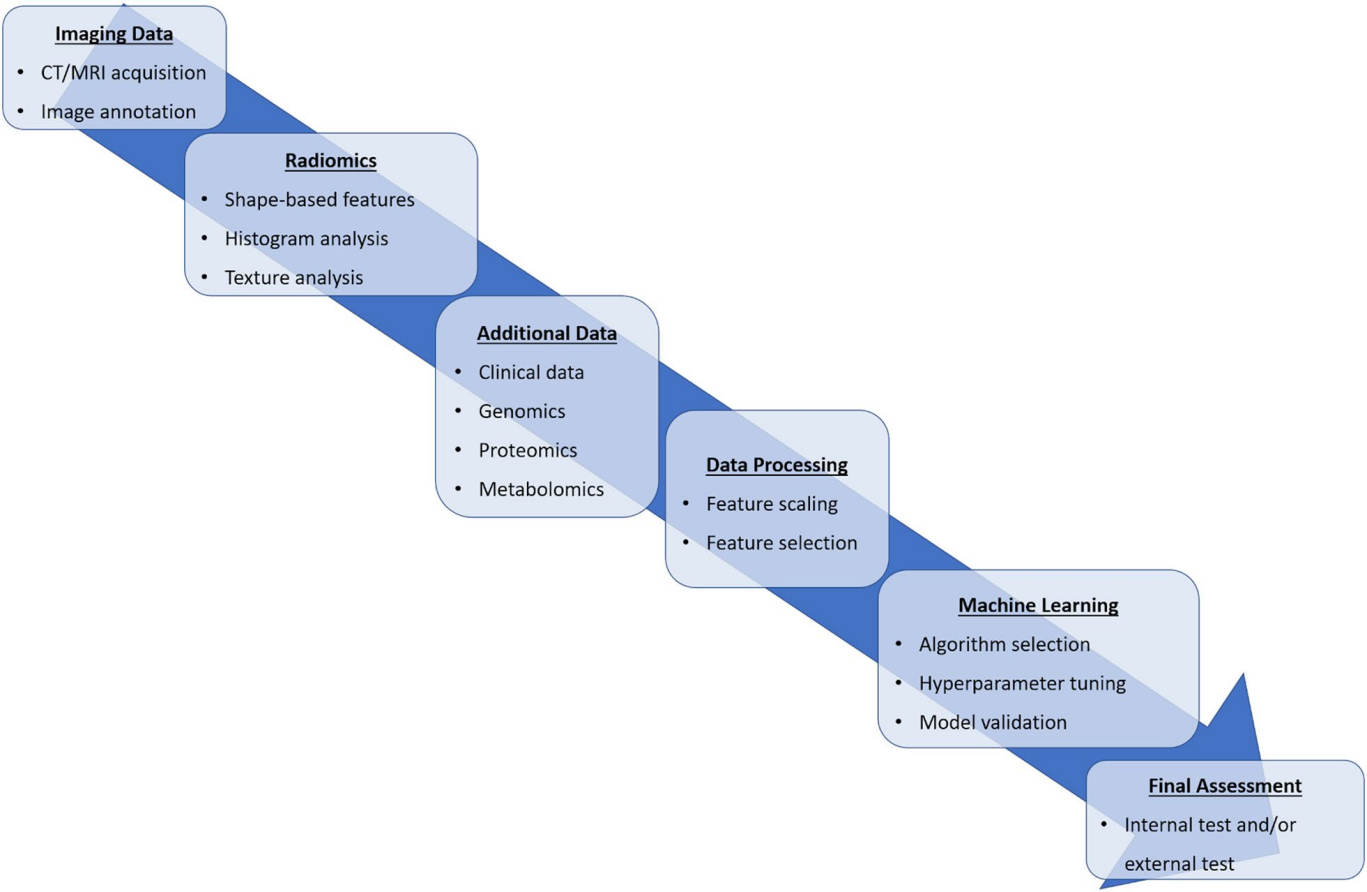
Example of a radiomics-based machine learning pipeline, listing the most commonly employed steps in an ideal order of execution

At least one machine learning validation technique was used in 25 (51%) of the 49 papers. K-fold cross-validation was used in most of the studies [13, 25, 28, 31–33, 37, 38, 40, 43, 44, 46–50]. The following machine learning validation techniques were used less commonly: bootstrapping [42, 51]; leave-one-out cross-validation [34, 35, 41]; leave-p-out cross-validation [52]; Monte Carlo cross-validation [23]; nested cross-validation [25, 27]; random-split cross-validation [20]. Figure 2 provides an overview of machine learning validation techniques. Figure 3 illustrates an example of a radiomics-based machine learning pipeline.

### Clinical validation

A clinical validation of the radiomics-based prediction model was reported in 19 (39%) of the 49 papers. It was performed on a separate set of data from the primary institution, i.e., internal test set, in 14 (29%) studies [15, 16, 22, 24, 28, 31, 32, 35, 37, 38, 41, 46, 47, 52]. It was performed on an independent set of data from the primary institution (related to a different scanner) or from an external institution, i.e., external test set, in 5 (10%) studies [25, 27, 29, 43, 51].

## Discussion

This systematic review focused on the radiomics literature regarding MRI and CT of bone and soft-tissue sarcomas with particular emphasis on reproducibility and validation strategies. The number of papers reporting the assessment of radiomic feature reproducibility and the use of independent or external clinical validation was relatively small. This finding is in line with recent literature reviews showing that the quality of sarcoma radiomics studies is low [53, 54], which may hamper performance generalizability of radiomic models on independent cohorts and, consequently, their practical application [53]. Thus, these issues need to be addressed in the radiomic workflow of future studies to facilitate clinical transferability.

### Baseline study characteristics

MRI and CT radiomics of bone and soft-tissue sarcomas have progressively gained attention in musculoskeletal and oncologic imaging. The number of papers has rapidly increased over the recent years, and almost half of those included in our review (47%) was published in 2020. Radiomics was used in attempt to answer clinical questions related to both diagnosis and prognosis of musculoskeletal sarcomas. Most studies (88%) were retrospective in nature, as this design allowed including relatively large number of patients with imaging data already available and bone or soft-tissue sarcomas, which are rare diseases. A prospective analysis, while not strictly necessary in radiomic studies [5], may, however, have advantages for controlling data gathering in reproducibility assessment and matching certain patient or imaging characteristics in independent datasets. Public data were used in no study regarding bone sarcomas and in a small proportion of the studies (6%) concerning soft-tissue sarcomas. A public database [55] available on The Cancer Imaging Archive (https://www.cancerimagingarchive.net) was used in all these studies. Public databases afford opportunities for researchers who do not have sufficient data at their institution and allow research groups from around the world to test and compare new radiomic methods using common data. Thus, research employing radiomics in this field would certainly be enhanced if further imaging databases are made publicly available in the near future.

Regarding segmentation, the process was performed manually in most of the studies (92%) and semiautomatically in the remaining, both requiring human intervention to some extent. Even though the influence of inter-observer and/or intra-observer variability on the reproducibility of radiomic features can be assessed as part of the radiomic workflow, fully automated segmentation algorithms would ideally achieve higher reliability and deserve future investigation. Annotations included the entire lesion volume (3D segmentation) in most of the studies (71%) and a single slice (2D), without multiple sampling, in the remaining (23%). However, to date no study has compared the outcome of 2D and 3D segmentations in musculoskeletal sarcomas. As 2D annotations are time saving and have recently proven higher performance than 3D segmentation in oropharyngeal cancers [56], this should represent another area of research in the near future. Of note, a limited number of studies (6%) used a 2D segmentation style with multiple sampling as a data augmentation technique to increase the number of labeled slices [26, 48, 57]. This practice can be useful for an uncommon entity as musculoskeletal sarcomas but should be employed with care to avoid the introduction of bias in the final model. The inclusion of samples from the same case in both the training and test sets could lead to overly optimistic results.

### Reproducibility strategies

A great variability in radiomic features has emerged as a major issue across studies and attributed to different segmentation, image acquisition, and post-processing approaches [4]. Therefore, methodological analyses are advisable prior to conducting radiomic studies in order to assess feature robustness and avoid biases due to non-reproducible, noisy features. This concept is in line with recent literature emphasizing the importance of reproducibility in artificial intelligence and radiology [58]. In our review, we noted that about one third of the included papers described a reproducibility analysis in their workflow. In this subgroup of papers, inter- and/or intra-reader segmentation variability was the main focus of the reproducibility analysis. Segmentation variability-related analyses outnumbered those addressing reproducibility issues due to image acquisition or post-processing differences, which were reported in one paper per each [30, 31]. This finding underlines that further research should deal with dependencies of radiomic features on image acquisition and post-processing. While these analyses may already be performed in retrospective series, when patients underwent more than one study in a short interval, prospective studies could facilitate the identification of reliable radiomic features within this domain. Finally, ICC was the statistical method used in most of the papers evaluating radiomic feature reproducibility. Of note, guidelines for performing and assessing ICC are available and can be followed to achieve consensus on the cutoff and threshold values [59].

### Validation strategies

Proper validation of radiomic models is highly desirable to bridge the gap between concepts and clinical application [53]. Machine learning validation techniques are employed to avoid any information leak from the test to the training set during model development [60]. Resampling strategies can be extremely useful, especially with relatively limited samples of data, which may not be truly representative for the population of interest, with the aim of reducing overfitting and better estimating the performance of the radiomics-based predictive model on new data (i.e., the test set) [61, 62]. K-fold cross-validation was the most commonly used technique for this task in the studies included in this review.

Ideally, in both prospective and retrospective studies, a clinical validation of the model is performed against completely independent sets of data, i.e., the external or independent test set [4]. We found that clinical validation was performed against an independent dataset from the primary institution (using different scanners) or from a different institution only in a small number of studies (10%) included in this systematic review. More studies (29%) validated the model using a separate set of data from the primary institution, i.e., an internal test set. Therefore, future studies should be carried out in more than one institution and include external testing of the model with large and independent sets of data.

## Limitations and conclusions

This study is limited to a systematic review of the literature, and no meta-analysis was performed due to the lack of homogeneity between studies in terms of objectives and subgroups of sarcoma with a rather limited number of papers per each objective and subgroup. Different metrics were also used, preventing us from providing an estimation of model performance for each objective. Furthermore, it was outside of the scope of the review to perform a formal assessment of the quality of each included study, as our focus was on reporting methodological data that can be in and of themselves quality indicators. Limitations notwithstanding, we reviewed the radiomics literature regarding bone and soft-tissue sarcomas with emphasis on the methodologic issues of feature reproducibility and predictive model validation. They varied largely among the included studies, and, in particular, no reproducibility analysis was provided in more than half the papers. Additionally, less than half the studies included a clinical validation, and only 10% used an independent dataset for this purpose. Thus, in order to bring the field of radiomics from a preclinical research area to the clinical stage, both these issues should be addressed in future studies dealing with musculoskeletal sarcomas.

## Data Availability

Not applicable.

## Abbreviations

CT: Computed tomography
ICC: Intraclass correlation coefficient
MRI: Magnetic resonance imaging
PRISMA: Preferred Reporting Items for Systematic reviews and Meta-Analyses
